# Actinomycosis of the Tongue: A Case Report and Review of Literature

**DOI:** 10.3390/antibiotics9030124

**Published:** 2020-03-16

**Authors:** Fiorella D’Amore, Roberto Franchini, Laura Moneghini, Niccolò Lombardi, Giovanni Lodi, Andrea Sardella, Elena M. Varoni

**Affiliations:** 1 UOC Odontostomatologia II, ASST Santi Paolo e Carlo—Presidio Ospedaliero San Paolo, 20142 Milano, Italy; fiura@hotmail.it (F.D.); rohfranchini@gmail.com (R.F.); 2 UOC Anatomia Patologica, Citogenetica e Patologia Molecolare; ASST Santi Paolo e Carlo—Presidio Ospedaliero San Paolo, 20142 Milano, Italy; laura.moneghini@asst-santipaolocarlo.it; 3 Dipartimento di Scienze Biomediche, Chirurgiche ed Odontoiatriche, Università degli Studi di Milano, 20142 Milano, Italy; niccolo.lombardi@unimi.it (N.L.); giovanni.lodi@unimi.it (G.L.); andrea.sardella@unimi.it (A.S.)

**Keywords:** tongue actinomycosis, oral actinomycosis, tongue lesion, oral infection, oral lesion

## Abstract

*Background*: Actinomycosis of the tongue is an uncommon, suppurative infection of lingual mucosa, caused by actinomyces. The clinical diagnosis may present serious difficulties because of its ability to mimic other lesions, including both benign and malignant neoplasms. *Methods*: Here, we describe the case of a 52-years-old patient affected by an asymptomatic, tumor-like tongue swelling, then diagnosed as actinomycosis. A review of tongue localization of actinomycosis is also reported, with emphasis on clinical findings and therapy. *Results and Conclusion*: Early diagnosis and treatment, with pus drainage and systemic antibiotic therapy, are pivotal to avoid severe and life-threatening complications.

## 1. Introduction

Actinomycosis is an uncommon, chronic, suppurative bacterial infection affecting soft-tissues caused by an anaerobic, filamentous Gram-positive bacterium, *Actinomyces israelii*. It was recognized for the first time in humans in about 1845 by Von Langenbeck [1].

Actinomycosis is classified by location as cervicofacial (50–60%), abdominal (25%), or pulmonary (15%) lesions. Cervicofacial actinomycosis is the most frequent clinical form, and the “lumpy jaw syndrome”, associated with odontogenic infection, is the most common clinical manifestation [2]. In the oral cavity, the infection commonly occurs in the mandibular area (submandibular region, ramus, and angle) in over 50% of cases [3,4], followed by cheek, parotid gland, tongue, nasal cavity, hypopharynx, gingival and oral space. Actinomycosis is associated with large abscesses and/or mandibular osteomyelitis, with or without the presence of the sinus tract [2]. Actinomyces spp. usually have a low pathogenicity and cause disease in a condition of poor oral hygiene, after surgical procedures and/or dental treatments such as extraction of a mandibular molar or as a complication of maxillofacial trauma.

In 1885, Von Hacker described the first case of actinomycosis of the tongue [5], which remains a very uncommon lesion of the oral cavity, representing less than 3% of all reported cases of actinomycosis in English literature [6,7]. Although it is a benign condition, if not treated, it can give, in later stages, osteomyelitis with extensive bone destruction [8,9]. Actinomycosis of soft tissues may extend to adjacent bone in up to 15% of cases [10,11].

Actinomycosis especially affects male patients aged 20 to 60 years [12]. The main predisposing factors include diabetes, alcohol abuse, malnutrition, and radiation therapy and immunosuppression [3]. Furthermore, patients affected by human immunodeficiency virus (HIV) had often shown to have poor outcomes [13,14].

The diagnosis of actinomycosis of soft tissues is based on clinical findings and on histological examination and staining of the lesion. The presence of sulphur granules in tissue sample or the pus drainage help during diagnosis, although nocardiosis may also present with sulphur granules [15,16,17]. The clinical diagnosis of tongue actinomycosis may present difficulties because of its ability to mimic other lesions, including both benign and malignant neoplasms. The histopathological examination is decisive to arrive to the final diagnosis and exclude other diseases.

Here, we describe the case of a patient who presented with a tumor-like lingual swelling, then diagnosed as actinomycosis of the tongue. A review of lingual actinomycosis cases is also reported.

## 2. Case Report

A 52-year-old man was presented at our clinical unit (Oral Medicine Unit, Odontostomatologia II, ASST Santi Paolo e Carlo, Presidio Ospedaliero San Paolo) because of an asymptomatic, not-ulcerated swelling of the anterior part of the tongue. The patient reported a recent occurrence of the lesion. In his clinical history, the patient declared that, about 5 years before, a biopsy was performed approximately at the same location of the current lesion, with a diagnosis of benign squamous papilloma. He did not report any previous history of local trauma or abdominal, thoracic, or cervical abscesses.

**Figure 1:**
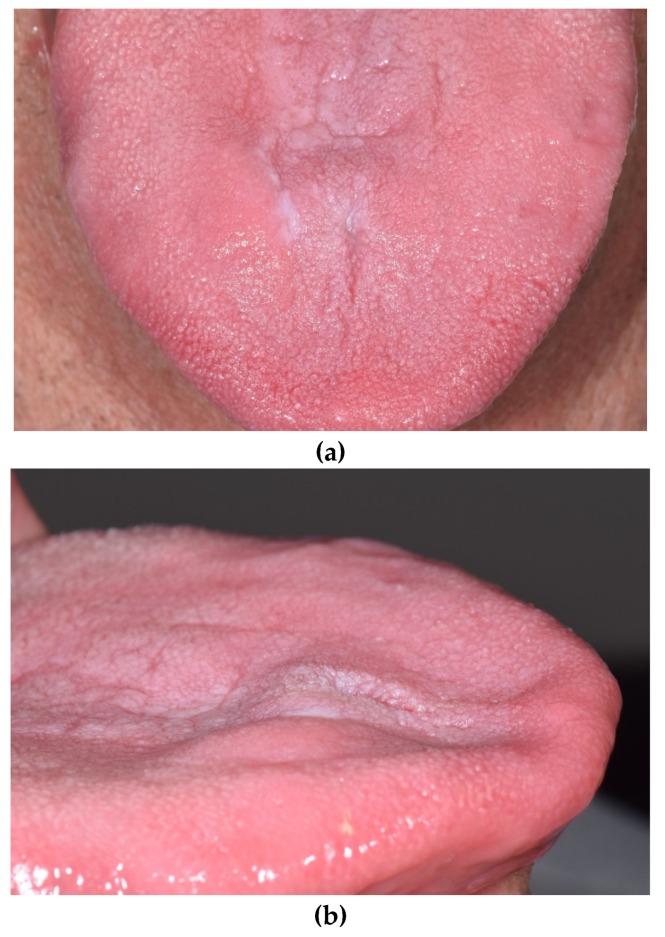
Tongue nodular lesion, as observed at first examination: the localized swelling on the lingual dorsum was covered by normal mucosa. (**a**) Frontal view of the lesion, (**b**) lateral view of the lesion.

Intraoral examination revealed a solid, fixed, bilobed submucosal nodule, located slightly on the right to the midline of the anterior part of the tongue (Figure 1a,b).

The lingual mucosa appeared normal in color, with two flat whitish spots and a small rounded erosion (about 2 mm in diameter). There was no restriction in tongue movement. Extraoral examination showed no evidence of cervical lymphadenopathy.

Hematological examination revealed neutrophilia, monocytosis, and a raised value of c-reactive protein (CRP), with a concentration of 3.5 mg/dL (normal range <0.5 mg/dL).

The clinical diagnosis was of a benign neoplasm, with a differential diagnosis including traumatic lesion, infective lesion or malignant neoplasm.

Under local anesthesia, two incisional biopsies of the lesion were taken for achieving the histopathological diagnosis—the first one of the epithelial layer and the other of the submucosa—in order to obtain a representative section. During the biopsy, a yellowish purulent drainage coming from the lesion was observed.

Three weeks after the biopsy, the patients received systemic antibiotic therapy because of a dental extraction. He was treated with oral intake of clarithromycin for one week (500 mg every 12 h), due to penicillin allergy.

**Figure 2:**
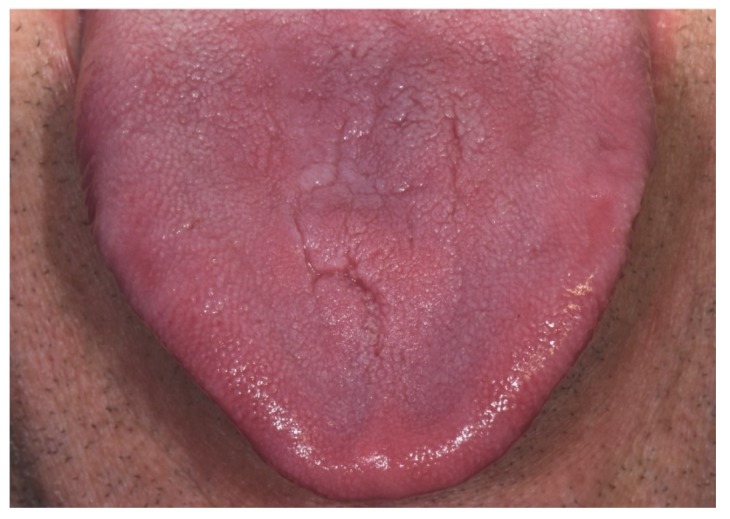
Complete resolution of the lesion at six weeks, after incisional biopsy with pus drainage and one-week antibiotic therapy.

Post-surgical healing was excellent, with the complete resolution of swelling at six weeks follow-up, when the histological result was given to the patient (Figure 2).

**Figure 3:**
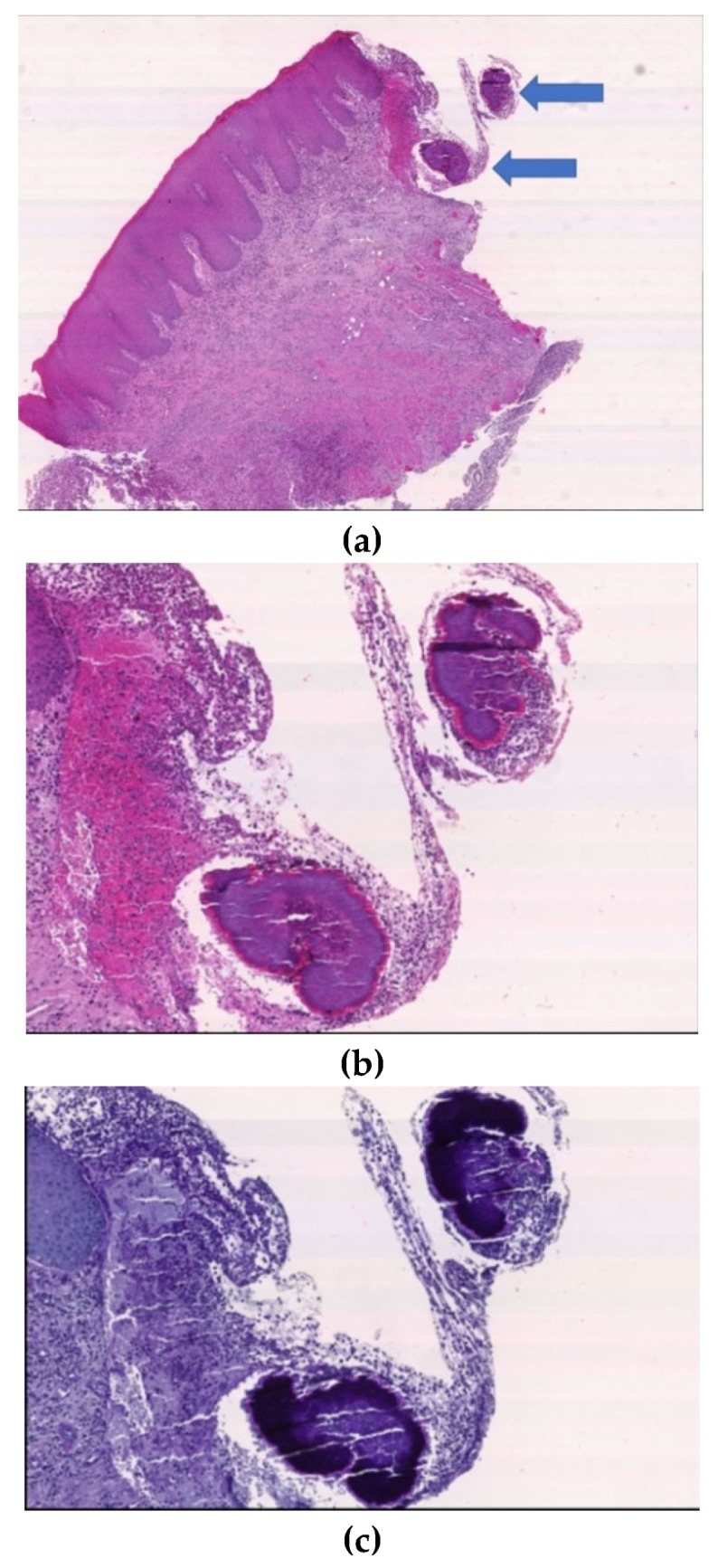
Histopathological examination of the lesion. At small magnification (20×; H&E) the oral mucosa shows acute and chronic intense inflammatory infiltration and, at one side of the sample, two aggregates (**a**) better assessed at high magnification (60×) (**b**) and after PAS staining (**c**) consistent with Actinomyces spp. colonies. The colonies are composed of Actinomyces spp. filaments, called “sulfurs granules”, surrounded by neutrophilic granulocytes.

Histopathological examination of the biopsy specimen, indeed, showed a nodular mass deep into the muscularis propria of the tongue, showing colonies of actinomyces and a chronic and acute inflammatory process, including suppurative signs (Figure 3).

## 3. Discussion

Lingual actinomycosis is rare. In Table 1, case reports/series available from the literature are reviewed, with particular attention to patient management [4,6,18,19,20,21,22,23,24,25,26,27,28,29,30].

In humans, the etiological agent of this disease is mainly *Actinomyces israelii*, a filamentous Gram-positive bacilli, commensal of the flora of the oropharynx, gastrointestinal tract and urogenital tract. In the oropharynx, it is particularly present in gingival sulcus, tonsillar crypts, dental plaques, and periodontal pockets. Consequently, actinomycosis is considered an endogenous infection, where the trauma plays a catalytic role in starting the disease [6,7,29].

The microorganism is unable to penetrate healthy tissue, while, to become invasive, it requires the interruption of mucosal barrier to gain access to the submucosal tissue [31]. Actinomycosis of the tongue is an uncommon disease since lingual epithelial layer has a structure particularly resistant to local trauma and infection. The keratinized mucosal lining, vascular-rich parenchyma, great mobility, and mechanical cleansing by saliva make difficult for bacteria to adhere and multiply.

Lingual actinomycosis is generally localized on the anterior two-thirds of the tongue, lateral to the medline [4], as occurred in this patient. When, after the trauma, such as self-biting (Table 1), the microorganism spreads deeply into the tissues and produces a massive fibrotic reaction surrounding the center of the lesion. Clinically, lingual actinomycosis appears as a hard nodular mass or swelling, slightly mobile on the adjacent layers, which can be rarely ulcerated and associated with necrotic tissue (Table 1). Although we could not retrieve specific information regarding recent traumas of the dorsum of the tongue, the patient reported a previous biopsy (with a diagnosis of squamous papilloma) at the same site of the current nodular lesion, which might have played a role in triggering the actinomycosis.

Pain, dysphagia, speech impairment, difficulty in moving the tongue can be reported by the patient (Table 1), although, in this report, the lesion was asymptomatic.

The gold standard for the final diagnosis is the histological examination including the histological staining to detect Actinomyces spp. colonies, whenever possible, also performed on purulent material. Bacterial culture is not recommended because it remains sterile in just about 50% of cases [2]. Typical microscopic findings include identification of Actinomyces spp. colonies and sulphur granules that are made of Gram-positive conglomeration of bacteria trapped in biofilm [2]. In our case, the diagnosis was achieved by the clinical findings and the identification of Actinomyces spp. colonies within the epithelial specimen.

The differential diagnosis should include other infections (lingual abscess, nocardiosis, botryomycosis), granulomatous lesions, infected cyst, pyogenic abscess, and benign and malignant neoplasms [29,30,31].

The preferred treatment remains administration of antibiotics with surgical excision or incision of the lesion. The drainage of abscess or surgical excision, when the lesion is small, largely enhance the efficacy of antibiotic therapy [29]. The drug of choice is penicillin. The addition of beta-lactamase inhibitors or metronidazole gives benefits with recurrent and polymicrobial Actinimycosis infections [32]. Other therapies include administration of third-generation cephalosporin or, in case of patients allergic to amoxicillin, macrolides and clindamycin [2,3]. In literature, there is no agreement on the ideal duration of therapy. Some studies suggested the need of long therapies, from weeks to months. Recurrence may occur after the cessation of the antibiotic [30], especially when the therapy was incomplete or with insufficient duration. No studies described local or distant recurrence of lingual actinomycosis after being treated successfully with drainage and a complete cycle of antibiotics (Table 1). In this case, the patient showed complete resolution of the clinical picture, lasting one month from the biopsy with pus drainage and antibiotic 1-week treatment with clarithromycin.

## 4. Conclusions

Although lingual actinomycosis infections is a rare event, early diagnosis and treatment are pivotal to avoid severe and life-threatening cases. Because of its ability to mimic other diseases, especially neoplasms, actinomycosis can be a challenging problem for the clinician and always requires diagnostic investigations, including biopsy.

This infection must be considered in the differential diagnosis of any cervicofacial mass. Treatment should always include pus drainage and systemic antibiotic therapy.

## Figures and Tables

**Table 1:** Studies reporting cases of actinomycosis of the tongue.

Reference	Oral Site (Number of Patients)	Clinical Appearance	Symptoms	Triggering Factors	Other Associated Signs	Treatment
SADEGHI, 2018 [24]	Tongue	Macroglossia	No	No	Dysphagia and speech impairment	Penicillin for 22 weeks
ANEJA, 2017 [28]	Tongue	Nodular mass	No	Tongue bite one month before	Difficulty in speech	Excision and amoxicillin–clavulanic acid for 2 weeks
JAT, 2017 [27]	Tongue	Nodular mass	No	No	Poor oral hygiene and dental caries	Excision. Doxycycline for 3 weeks
ROCHA, 2017 [26]	Tongue	Necrotic tissue with purulent right lateral tongue developed after sclerosing agent injection to treat vascular lesion	No	No	No	Local debridement
ESCODA, 2013 [23]	Tongue	Two ulcerated lesions	Pain	No	Erosive lichen asymptomatic	Clyndamicin (unknown posology) and chlorhexidine digluconate for 1 month
KURTARAN, 2011 [22]	Tongue	Solid mass	Pain	Dental prosthesis problem 1 month before	Dysphagia and speech impairment	Excision and combination of amoxicillin-clavulanic acid and metronidazole for 5 weeks
HABIBI, 2008 [31]	Tongue	Solid mass	No	Tongue bite	No	Excision and intravenus penicillin for 3 weeks
ATESPARE, 2006 [7]	Tongue	solid mass	No	No	Speech disturbance	Excision and amoxicillin-clavulanic acid for 3 weeks
VAZQUEZ, 1997 [29]	Tongue	Swelling	No	No	No	Amoxicillin for 4 weeks
GERBINO, 1996 [4]	Tongue (*n* = 2)	Nodules	NA	NA	NA	Penicillin V for 1 month
FICARRA, 1993 [31]	Tongue	Swelling	No	No	No	Penicillin for 2 weeks
ISALSKA, 1991 [21]	Tongue	Swelling	Local discomfort	No	No	Amoxycillin for 6 month
BRIGNALL, 1989 [19]	Tongue	Swelling	Acute discomfort	Accidental self-inflicted bite to the tongue 6 months previously	Lost normal movement and dysphagia	Henoxy-methyl-penicillin for 3 month
KUEPPER, 1979 [6]	Tongue	Mass	NA	NA	NA	Penicillin for 1 month
UHLER, 1972 [25]	Tongue	Nodular mass	NA	NA	NA	Excision and penicillin for 6 months
SODAGAR, 1972 [20] *	Tongue	Solid mass	NA	NA	NA	Excision

* Full-text not available, only abstract; NA = not available information.
